# A wavelet-based ECG delineation algorithm for 32-bit integer online processing

**DOI:** 10.1186/1475-925X-10-23

**Published:** 2011-04-03

**Authors:** Luigi Y Di Marco, Lorenzo Chiari

**Affiliations:** 1 Biomedical Engineering Group, Department of Electronics, Computer Science and Systems (DEIS), University of Bologna, I-40136 Bologna, Italy

## Abstract

**Background:**

Since the first well-known electrocardiogram (ECG) delineator based on Wavelet Transform (WT) presented by Li *et al. *in 1995, a significant research effort has been devoted to the exploitation of this promising method. Its ability to reliably delineate the major waveform components (mono- or bi-phasic P wave, QRS, and mono- or bi-phasic T wave) would make it a suitable candidate for efficient online processing of ambulatory ECG signals. Unfortunately, previous implementations of this method adopt non-linear operators such as *root mean square *(RMS) or floating point algebra, which are computationally demanding.

**Methods:**

This paper presents a 32-bit integer, linear algebra advanced approach to online QRS detection and P-QRS-T waves delineation of a single lead ECG signal, based on WT.

**Results:**

The QRS detector performance was validated on the MIT-BIH Arrhythmia Database (sensitivity Se = 99.77%, positive predictive value P+ = 99.86%, on 109010 annotated beats) and on the European ST-T Database (Se = 99.81%, P+ = 99.56%, on 788050 annotated beats). The ECG delineator was validated on the QT Database, showing a mean error between manual and automatic annotation below 1.5 samples for all fiducial points: P-onset, P-peak, P-offset, QRS-onset, QRS-offset, T-peak, T-offset, and a mean standard deviation comparable to other established methods.

**Conclusions:**

The proposed algorithm exhibits reliable QRS detection as well as accurate ECG delineation, in spite of a simple structure built on integer linear algebra.

## Background

The electrocardiogram (ECG) is the recording of the electrical activity of the heart by means of electrodes placed on the body surface. It is the most commonly used non-invasive test in primary care for heart rate and rhythm-related abnormalities detection [1,2]. In recent years the interest for the ECG signal analysis has extended from clinical practice and research to disciplines such as cognitive psychophysiology [3,4], physical training [5,6] and rehabilitation [7].

Many non-diagnostic applications do not require the full 12-lead setup of clinical ECG, employing a limited number of electrodes. In some cases a single lead setup, requiring only three electrodes, is sufficient. Such applications focus on ambulatory ECG monitoring, namely in unconstrained conditions, in which subjects perform normal activities as in their daily life [4], [8-10].

Ambulatory ECG analysis requires processing of signals which are affected by considerable noise, mainly caused by electrode motion and muscular activity, more prominently than in resting ECG recordings, and by power-line coupling. Moreover, emerging wearable technologies for ambulatory ECG monitoring have limited processing resources and low power budget.

Clinical information on the cardiac beat is carried by the waveforms appearing on the electrocardiogram, namely: QRS-complex and P, T, U, waves. Their amplitudes and relative time intervals provide insight on heart rhythm abnormalities and heart disease such as ischemia and myocardial infarction. Electrocardiogram delineation is the automatic process of determining such amplitudes and time intervals.

Performing an accurate delineation is quite a challenging task, for many reasons. For example, the P wave is characterized by low amplitude and may be masked by electrode motion or by muscular noise. The P and T waves may be biphasic, which increases the difficulty to accurately determine their onset or offset. Moreover, some arrhythmic beats may not contain all the standard ECG waves, for example the P wave may be missing, while in accelerated heart rate patterns, it might be partially overlapped to the T wave of the previous beat.

The first stage of ECG delineation is devoted to detecting the QRS-complex, which in most cases is the most pronounced wave of the heart cycle. Subsequent processing locates P, QRS-complex and T waves fiducial points (onset, peak, offset).

The cyclic nature of the ECG signal and its spectral components, which mainly appear in well-known and distinguishable frequency bands, make ECG a suitable candidate for multi-resolution decomposition by means of wavelet transforms [11,12]. Methods based on wavelet transforms have been proposed by numerous authors [13-18], building on the first well-known ECG delineator proposed by Li *et al. *[19].

Unfortunately, most of these ECG delineation algorithms adopt non-linear operators such as *root mean square *(RMS) or floating point algebra, which are computationally demanding. The work by Sovilj *et al. *[17] presents a real-time implementation of QRS detection and P wave delineation, though no validation on standard databases is provided, nor is the P wave delineation criterion explained. In [20] a WT-based algorithm for real-time QRS detection and ECG delineation is presented, though no validation is reported on delineation, and the total number of annotated beats used in the validation of QRS detection does not match the record-by-record count, as noted in [13].

The work by Boichat *et al. *[16] presents a real-time implementation of the offline method proposed by Martinez *et al. *[13], though no validation on arrhythmia databases (such as the MIT-BIH Arrhythmia Database) is provided. The delineation of QRS onset and QRS offset in [16] is performed on WT detail coefficients at scale 2^4^, namely on the output of a pass-band FIR filter with a 3dB band of 4.1-13.5 Hz. Moreover, the criterion adopted for the validation of the delineation algorithm is based on a 320 ms window, which exceeds the maximum tolerance (150 ms) for QRS detection accuracy allowed by the ANSI/AAMI-EC57:1998 standard.

This paper presents a wavelet-based algorithm for single lead QRS detection and ECG delineation of P wave, QRS-complex and T wave, under the algorithmic constraint of 32-bit integer linear algebra online processing and compliance with ANSI/AAMI-EC57:1998 requirements on QRS detection accuracy. The algorithm was validated on MIT-BIH Arrhythmia Database (MITDB), the European ST-T Database (EDB), and QT Database (QTDB), available from Physionet.

## Methods

### Wavelet Transform

The general theory on wavelet transforms for multi-resolution analysis is described in detail in [11,12], [21] and its application to ECG signal delineation is presented in [13], [19], while a review is given in [14].

With reference to the family of spline functions of degree *2r + 2 *proposed in [12] for the smoothing function *θ(t)*, in this study the 8^th ^degree (*r *= 3) was adopted. Its Fourier transform is expressed in (1.1) and the Fourier transform of the wavelet function is expressed in (1.2).(1.1)(1.2)

Unlike previous studies [13], [16-20] where a cubic spline smoothing function *θ(t) *(*r *= 1) was used, in this study a higher value of *r *was adopted to reduce the width of the compact support and the pass-band of the equivalent filter for scales higher than 2^1^, to improve frequency band separation across scales. However, the number of filter taps increases with *r*, therefore a tradeoff should be determined between computational effort and delineation performance.

**Figure 1:**
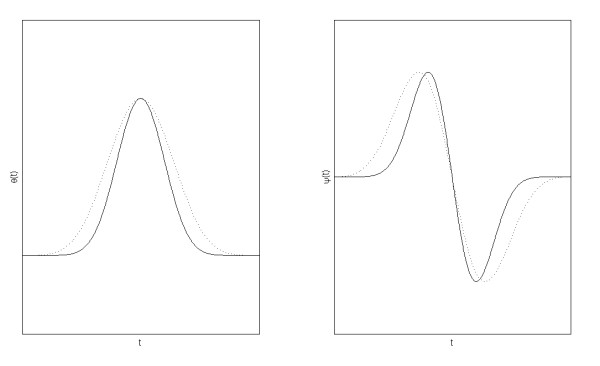
**Smoothing function and wavelet function**. Smoothing function *θ(t) *(left) and wavelet function *ψ(t) *(right), for *r *= 1 (dotted line) and *r *= 3 (solid line).

Figure 1 shows the smoothing function *θ(t) *and wavelet function ψ(t) for *r *= 1 and *r *= 3. The compact support of the smoothing (scaling) function decreases in width as *r *increases.

The low-pass filter *H *and high-pass filter *G *derived from (1.1) and (1.2) can be expressed as:(2)

whose finite impulse response *hn *and *gn *are given by the coefficients reported in Table 1.

**Table 1:** Wavelet Filters Impulse Response

*N*	*h*_*n*_	*g*_*n*_
-2	1/128	
-1	7/128	
0	21/128	-2
1	35/128	2
2	35/128	
3	21/128	
4	7/128	
5	1/128	

It shall be noted that *hn *is symmetrical and of even length, representing a linear phase low-pass FIR filter, while *gn *is anti-symmetrical of even length, representing a linear phase high-pass FIR filter.

The frequency response for the filter bank generalized for any given scale can be written as:(3)

**Figure 2:**
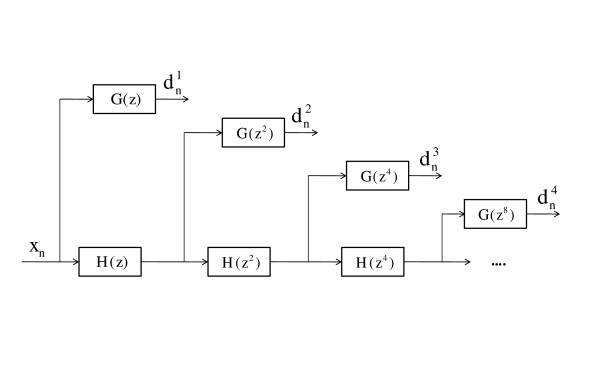
**DWT filter bank**. Filter bank implementation of biorthogonal dyadic wavelet transform without decimation (*algorithme à trous*). *d*^*k*^_*n *_is the detail coefficient series for scale 2^k^. Inspired by [13].

The filter bank structure is illustrated in Figure 2.

**Figure 3:**
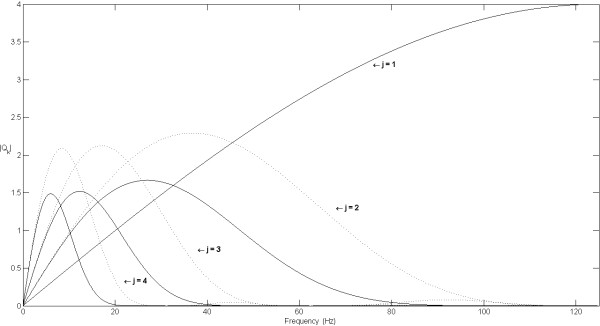
**Equivalent filters magnitude response**. Equivalent Filters Q_k _magnitude response, for different scales 2^k^, for *r *= 1 (dotted line) and *r *= 3 (solid line). Sample frequency *F*_*s *_= 250 samples/s.

The frequency response of the equivalent filters *Qk *in (3) is displayed in Figure 3 for the first four scales, for *r *= 1 (cubic spline smoothing function) and *r *= 3 (8^th ^degree spline smoothing function). For any given scale 2^k^, *Qk *pass-band narrows with increasing *r*, improving frequency separation of the filter bank across scales.

The group delay of the equivalent filter *Qk *must be accounted for in multi-scale analysis of discrete wavelet transform (DWT) coefficients. To match zero-crossings (and their relative modulus-maxima) across different scales, DWT coefficients must be aligned temporally.

The group delay of Q_k _at scale 2^k^, k >1, is given by:(4.1)

where:(4.2)

is the group delay of the low-pass filter at scale 2^l^, and(4.3)

is the group delay of the high-pass filter at scale 2^k^.

According to [22,23], the energy of the main waveforms composing the ECG, namely QRS-complex, P and T waves, lies within a limited frequency range. As a consequence, a limited number of scales is required for ECG delineation. Table 2 summarizes the cutoff frequencies of *Qk *filters for the scales of interest, for *r *= 1 and *r *= 3.

**Table 2:** Wavelet Filters Bandwidth

Scale	Bandwidth [Hz] (*)	Bandwidth [Hz] (*)
2^k^	3^rd ^degree Spline θ(t) (*r *= 1)	8^th ^degree Spline θ(t) (*r *= 3)
k = 1	62.50 - 125.00	62.50 - 125.00
k = 2	18.02 - 58.60	13.12 - 43.55
k = 3	8.36 - 27.46	5.98 - 19.99
k = 4	4.11 - 13.52	2.93 - 9.80

(*) 3 dB cut-off

### Description of the Algorithm

The raw ECG signal is assumed to be sampled at 250 samples/s.

The databases used for validation contain records of ECG data stored at 12-bit/sample. Therefore, to prevent overflow in a (signed) integer implementation of the low-pass filter adopted in the filter bank, 16-bit integer capacity is not sufficient. This constitutes the only reason for adopting a 32-bit instead of 16-bit implementation. However, a 32-bit implementation also complies with input signals (raw ECG data) with a sample resolution up to 24-bit/sample. Most, if not all, commercially available ECG front-end devices currently fall within this category. In order to comply with the largest set of such devices on the market, no assumptions are made on the amplitude resolution.

The DWT properties which the proposed method is based on are well described in [13], [19]. Based on the properties of the filter bank (2), the zero-crossings of the DWT coefficients *d*^*k*^_*n *_correspond to the local maxima or minima of the smoothed input signal at different scales, and the maximum absolute values of *d*^*k*^_*n *_are associated with maximum slopes in the filtered signal [13].

**Figure 4:**
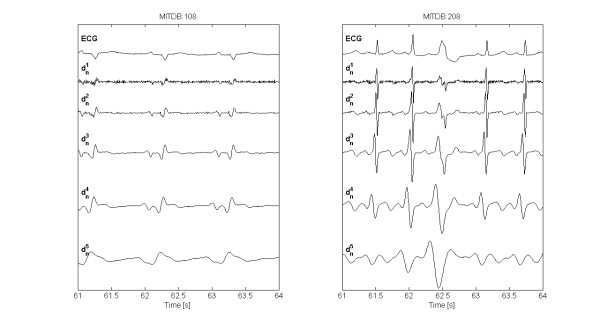
**ECG signal and DWT decomposition**. Examples of ECG signals from MITDB records (resampled at *F*_*s *_= 250 samples/s) MITDB:108 (left), MITDB:208 (right), and DWT detail coefficient *d*^*k*^_*n *_at scales 2^1 ^through 2^5^.

Figure 4 shows DWT detail coefficients computed by the present algorithm, for actual ECG signals (record 108 and 208, from MITDB).

At a sampling frequency of 250 samples/s, the spectral content of the ECG signal mainly falls within the first five scales of the filter bank (2). In particular, the QRS-complex is prominent at scales 2^2 ^and 2^3 ^while its energy decreases at increasing scales and becomes very low at scales higher than 2^4^, while P shows high energy at scale 2^3 ^which decreases at higher ones. At scales 2^3 ^through 2^5 ^T wave has high energy, though at scale 2^5 ^the baseline drift, including respiration effects, becomes prominent. For this reason, scale 2^5 ^is not considered in this study. At scales 2^1 ^and 2^2 ^small peaks in Q and S waves may show zero-crossings though at such low scales, especially scale 2^1^, muscular noise and power-line coupling may appear.

**Figure 5:**
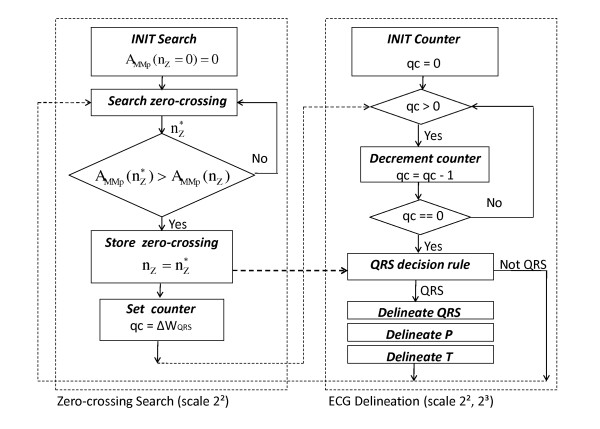
**ECG delineation state machine**. State Machine flow chart for online ECG delineation. The zero-corssing *n*_*Z *_with the largest modulus-maxima pair amplitude *A*_*MMp*_*(n*_*Z*_*) *is detected within ΔW_QRS _(250 ms) at scale 2^2^. Detail coefficients *d*^*k*^_*n *_at scales 2^2 ^and 2^3 ^are parsed for the verification of QRS candidates according to the QRS-detection decision rule. After QRS-complex detection, the delineation process delineates in the order: the QRS-complex, the P wave, and the T wave.

Using the information of local maxima, minima and zero-crossing at the scales of interest, the algorithm identifies for each beat the significant points of the ECG in the following steps: 1) detection of the QRS-complex; 2) QRS-complex delineation (onset, offset); 3) P wave delineation (onset, peak, offset); 4) T wave delineation (peak, offset) of the previous beat. Figure 5 displays the flow chart of the state machine for online parsing of detail coefficients *d*^*2*^_*n*_, for QRS detection. Unlike previous works [13], [19], for QRS detection only two scales (2^2^, 2^3^) are processed.

The algorithm proposed in this work is intended for online processing, therefore it is causal: at discrete time *Ti*, only ECG samples at *Tk *≤ *Ti *are assumed to be available.

To comply with low power budget constraints, the algorithm does not perform back-search for missed beats. The drawback is a decrease in sensitivity; the advantage is a decrease in storage memory and processing time. A memory buffer of 1 s for WT coefficients is sufficient for QRS detection, whereas the required storage size increases (depending on the inter-beat interval duration, in general no more than 1.5 s) for computing delineation of the T wave of the previous beat.

### QRS detection

For each beat, the QRS-complex is detected using wavelet detail coefficients *d*^*k*^_*n *_at scales 2^2 ^and 2^3^. As shown in Figure 5, only scale 2^2 ^is parsed for zero-crossings. When a zero-crossing is detected, the adjacent modulus-maxima pair *MMp(n*_*Z*_*) *is determined and the associated amplitude *A*_*MMp*_*(n*_*Z*_*)*, defined as the difference between the positive maximum and negative minimum detail coefficients, is computed. The zero-crossing is stored and an observation window Δ*WQRS *of 250 ms is initialized: if a new zero-crossing *n*_Z_^* ^is detected within this window such that *A*_*MMp*_*(n*_Z_^*^) > A_*MMp*_*(n*_*Z*_*)*, the window is reset and *n*_Z_^* ^is stored, replacing *n*_*Z*_, as shown in Figure 5. The process is iterated until a full window elapses without new candidates. The zero-crossing *n*_*Z *_represents the QRS-candidate. The QRS-detection decision rule is defined as follows: a window of 200 ms centred around *n*_*Z *_is considered, and the maximum-minimum difference *Δd*^*2*^_*n*_*(n*_*Z*_*) *of detail coefficients within such window, at scale 2^2^, is computed as follows:(5.1)

where ΔW_100 _represents a time interval of 100 ms expressed in units of samples. The following condition is then tested:(5.2)

where *ε*^*2*^_*QRS *_is an empirically determined threshold computed as follows:(5.3)

where the summation encompasses the N (= 4) most recent QRS-candidates that satisfied (5.2). Under the assumption that the time distance between two consecutive beats is generally not longer than 2 s (corresponding to a heart rate of 30 beats/min), it takes not more than 8 s to collect N (= 4) confirmed candidates. For this reason, a learning period of 8 s is allowed before the algorithm outputs any detected beats.

If (5.2) is met, the decision process proceeds to the next step considering scale 2^3^:(5.4)(5.5)

where *ε*^*3*^_*QRS *_is an empirically determined threshold computed as in (5.3), for scale 2^3^. It shall be noted that, in (5.4), *n *spans the same window as in (5.1). Coefficients across different scales are time-aligned by accounting for the group delay computed in (4.1).

If (5.2) and (5.5) are met, the QRS-candidate is confirmed, and thresholds *ε*^*2*^_*QRS *_and *ε*^*3*^_*QRS *_are updated. Then, if the learning period is expired, the zero-crossing is marked as the local peak (fiducial point) of a QRS-complex, and the algorithm proceeds for the delineation of P, QRS, T waves. It shall be noted that thresholds *ε*^*2*^_*QRS *_and *ε*^*3*^_*QRS *_are initialized to zero and iteratively adapt to QRS candidates. At the early stages of this process, QRS misdetections (false positives) are likely to occur. To prevent this, the algorithm does not output any detected QRS complexes until the learning period has expired. A learning period of 8 s is generally sufficient, although there may be extreme conditions such as lead-fail, cardiac arrest, poor signal-to-noise ratio, in which a longer time is required.

### QRS delineation

QRS delineation is performed at scale 2^2^. After detecting the QRS-complex, the QRS onset fiducial point is determined starting from the position *n*_*pre *_of the modulus maximum preceding the zero-crossing *n*_*Z *_of the QRS-complex at scale 2^2^.

The following thresholds are defined, based on local *d*^*2*^_*n *_coefficient values:(5.6)

where *n*_*post *_is the sample index of the modulus maximum following *n*_*Z*_. The delineation algorithm searches back from *n*_*pre *_for negative minima or positive maxima, and stores the first crossing of the threshold ε^2^_Qon, I _to be assigned to QRS onset in case no modulus maxima are found within a fixed size window of 120 ms preceding *n*_*pre*_.

The algorithm stops when a modulus maximum is detected whose amplitude is lower than the threshold *ε*^*2*^_*Qon,II*_, or the end of the search window has been reached. If at least one modulus maximum is found, a new threshold is defined:(5.7)

where *n*_*left *_is the sample index at which *d*^*2*^_*n *_has its left-most modulus maximum. The algorithm searches back from *n*_*left *_until the first crossing of the new threshold *ε*^*2*^_*Qon,III *_or the end of the fixed-size window is reached. The value is assigned to QRS onset. The symmetrical criterion is adopted for the determination of QRS offset, starting from the position *n*_*post *_of the modulus maximum following the zero-crossing *n*_*Z*_. The threshold used for QRS offset delineation are:(5.8)

where *n*_*right *_is the sample index of the right-most modulus maximum following *n*_*post *_whose amplitude exceeds threshold *ε*^*2*^_*Qoff,II*_.

**Figure 6:**
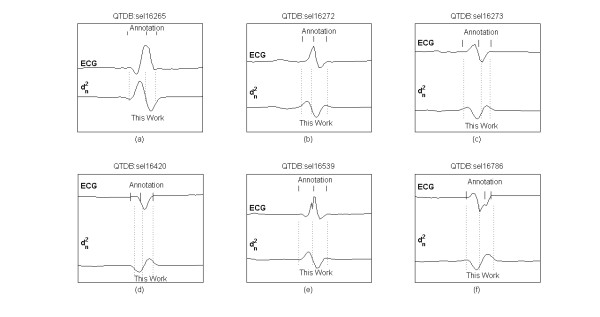
**Delineation of QRS morphologies**. Examples of various QRS morphologies from QTDB records, with manual annotations (top) and delineated characteristic points (bottom): QRS onset, dominant QRS peak, QRS offset.

Figure 6 shows examples of different QRS morphologies from QTDB records, the related manual annotations and the automatic delineation markers.

### P wave delineation

P wave delineation is performed at scale 2^3^. Mono- and bi-phasic P waves are handled. After delineating the QRS-complex, the algorithm searches back from QRS onset on scale 2^3 ^for the P wave. A fixed-size window whose length is chosen to be the shortest between 300 ms and half the last inter-beat interval is used for the search. Within this window, all zero-crossings are stored. The zero-crossing search is limited to a sub-portion of the window excluding the first (left-most) 100 ms which are only used for determining P onset. The zero crossing *n*_*Z *_with maximizes *A*_*MMp*_*(n*_*Z*_*) *is marked as P wave dominant peak. To determine the mono- bi-phasic morphology of the P wave, specific conditions are tested:(6.1)

where |*d*^*3*^_*n pre*_| is the modulus maximum preceding the zero-crossing *n*_*Z*_, at scale 2^3^, and |*d*^*3*^_*n post*_| is the modulus maximum following n_Z_. If (6.1) is verified, and a zero-crossing *n*^*L*^_*Z *_preceding *n*_*Z *_is available within a distance of 100 ms, (6.1) is tested also for *n*^*L*^_*Z*_. If such condition is verified, the following is also tested:(6.2.1)

If (6.1) and (6.2.1) are verified for *n*^*L*^_*Z*_, the P wave is considered to be bi-phasic and *n*_*pre *_is defined as the sample corresponding to the left-most modulus maximum of *MMp(n*^*L*^_*Z*_*) *otherwise *n*_*pre *_is defined as the sample corresponding to the left-most modulus maximum of *MMp(n*_*Z*_*)*.

The same procedure is adopted in the search of *n*^*R*^_*Z *_following *n*_*Z *_within a distance of 100 ms. If (6.1) is verified for *n*^*R*^_*Z*_, the following condition is tested:(6.2.2)

If (6.1) and (6.2.2) are verified for *n*^*R*^_*Z*_, the P wave is considered to be bi-phasic and *n*_*post *_is defined as the sample corresponding to the right-most modulus maximum of *MMp(n*^*R*^_*Z*_*) *otherwise *n*_*post *_is defined as the sample corresponding to the right-most modulus maximum of *MMp(n*_*Z*_*)*.

The sample *n*_*pre *_becomes the starting point for searching back the first crossing of a threshold:(6.3)

If such crossing point is found within the search window, it is assigned to P onset.

The algorithm then searches for P offset, namely the estimated end of P, adopting the same procedure described for P onset. The threshold adopted is:(6.4)

If P onset, peak and offset are found within the search window, P wave delineation result is positive, otherwise the algorithm declares that P wave could not be delineated for the given beat.

**Figure 7:**
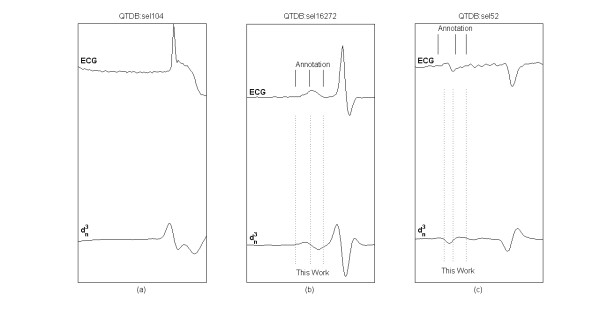
**Delineation of P wave**. Examples of P waves from QTDB records, with manual annotations (top) and delineated characteristic points (bottom): P onset, dominant P peak, P offset. (a) absent P wave, (b) positive P wave, (c) bi-phasic P wave.

Figure 7 shows examples of P morphologies from QTDB records, the related manual annotations and the automatic delineation markers.

### T wave delineation

T wave delineation is performed at scale 2^3^. The following possible morphologies are handled: positive (+), negative (-), biphasic (+/- or -/+), upward and downward. At each identified QRS-complex, T wave is delineated for the previous beat. The search is done over a window defined as:(7.1)

where *n*_*QRS off (i-1) *_denotes the sample of the QRS offset of the previous beat (assuming the i*th *beat is the latest detected), *rr *is the distance in units of samples between the i*th *and the (i-1)*th *QRS fiducial point, and ΔW_80 _represents an interval of 80 ms expressed in units of samples. The T wave dominant peak is searched within a sub-window of ΔW_T_:(7.2)

Within ΔW_T PK _all zero-crossings are stored. A zero-crossing *n*_*Z *_is considered to have a positive (negative) *slope *if the first non-zero detail coefficient preceding *n*_*Z *_is negative (positive), and the first non-zero detail coefficient following *n*_*Z *_is positive (negative). For zero-crossings *n*_*Z *_with negative (positive) slopes, the maximum (minimum) value *M*_*n pre *_of positive (negative) *d*^*3*^_*n *_coefficients preceding *n*_*Z *_is stored, together with the minimum (maximum) value *M*_*n post *_of negative (positive) *d*^*3*^_*n *_coefficients following *n*_*Z*_. The absolute value of the difference *Δ*_*MM*_*(n*_*Z*_*) *between *M*_*n pre *_and *M*_*n post *_is computed and the zero-crossing *n*_*Z *_with the highest value is considered. If an adjacent zero-crossing *n*^*L*^_*Z *_to the left of *n*_*Z *_exists and the following condition is met:(7.3)

then the T wave is considered biphasic, *n*^*L*^_*Z *_is marked as T wave dominant peak *T*_*pk*_, *n*_*Z *_is marked as the end *T*_*off *_of the dominant wave (i.e. the wave whose peak is surrounded by the largest slopes), and the bi-phasic T wave end *T*_*end *_is searched to the right of *n*_*post *_following *n*_*Z*_. *T*_*end *_is then assigned to the first sample for which *d*^*3*^_*n *_falls below a threshold *ε*^*3*^_*Tend *_defined as:(7.4)

If *n*^*L*^_*Z *_does not exist or (7.3) is not verified, *n*_*Z *_is marked as *T*_*pk*_, and the search proceeds to the right of *n*_*post *_following *n*_*Z*_. *T*_*off *_is assigned to the first sample for which *d*^*3*^_*n *_falls below a threshold *ε*^*3*^_*Toff*_, defined as;(7.5)

where *n*_*post *_refers to *n*_*Z*_. If an adjacent zero-crossing *n*^*R*^_*Z *_exists to the right of *n*_*Z*_, such that:(7.6)

the T wave is considered to be bi-phasic and *T*_*end *_is defined as the first sample for which *d*^*3*^_*n *_falls below the threshold in (7.4) where *n*_*post *_now refers to *n*^*R*^_*Z*_.

**Figure 8:**
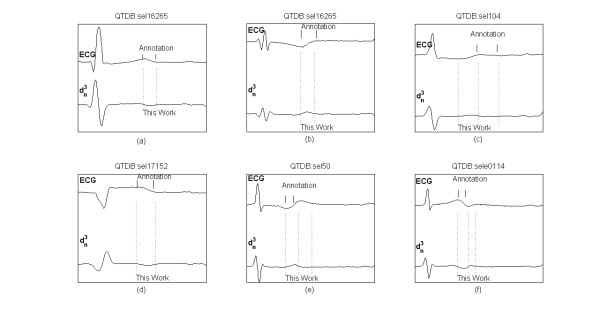
**Delineation of T wave**. Examples of T waves from QTDB records, with manual annotations (top) and delineated characteristic points (bottom): dominant T peak, dominant T offset, T end. (a) positive T wave, (b) negative T wave, (c) upward T wave, (d) downward T wave, (e) and (f) bi-phasic T wave.

Figure 8 shows examples of various T wave morphologies from QTDB records, the related manual annotations and the automatic delineation markers.

### Validation

The QRS detection algorithm was validated on manually annotated ECG databases, namely the MIT-BIH Arrhythmia Database (MITDB) and the European ST-T Database (EDB), whereas the P-QRS-T delineation algorithm was validated on the QT Database (QTDB).

The MITDB database includes a selection of Holter recordings covering a broad spectrum of arrhythmias.

The EDB database contains annotated excerpts of ambulatory ECG recordings with a representative selection of ECG abnormalities including ST segment displacement and cardiac axis shifts.

The QTDB database contains records from MITDB and EDB, and from several other databases (Normal Sinus Rhythm, ST Change, Supraventricular Arrhythmia, Sudden Death, Long Term Recordings). This database was created for validation of waveform boundaries and contains annotations by cardiologists for at least 30 beats per record, including QRS-complex, P, T, U waves delineation.

For the QRS detector validation on MITDB and EDB, the first ECG channel was used and, for MITDB only, raw data were resampled at 250 samples/s before processing.

For the validation on QTDB, reference annotations of first cardiologist (*q1c *files from QTDB) were used in this work. Records from this database are sampled at 250 samples/s, therefore no resampling was required.

Table 3 summarizes the databases used for validation.

**Table 3:** Databases used for validation

Database	#Annotated Beats	Records	Record Duration
MITDB	109010	48	30 min
EDB	788050	90	120 min
QTDB	3622	105	15 min

To assess QRS detection performance, sensitivity (*Se*) and positive predictive value (*P*^+^) were calculated: *Se *= TP/(TP+FN) where TP is the total number of true positives identified in the given record, FN is the total number of false negatives; *P*^+ ^= TP/(TP+FP) where FP is the total number of false positives.

A true positive is achieved when the time difference between the given annotated beat and the detected beat is not greater than 150 ms, in compliance with ANSI/AAMI-EC57:1998 standard.

For the validation of ECG delineation on QTDB, the metrics proposed in [13], [16] was adopted, where *m *is the mean value of the errors intended as the time difference between automatic and reference annotation, for all annotations, and *s *is the average standard deviation of the error, calculated by averaging the intra-recording standard deviations.

For each fiducial point delineation, the ECG channel with the least error was chosen, as in [13], [16]. Sensitivity was calculated for each characteristic point, for P wave, T wave and QRS-complex, separately. For T wave, manual annotations T-peak and T-offset, are matched to *T*_*pk *_an *T*_*off *_as defined in the delineation method, respectively.

A true positive is achieved when the wave is annotated and the delineation process detects the presence of such wave within a time distance not greater than 150 ms. (in [16] a window of 320 ms is used, in [13] the window size is not reported). A false positive occurs when the delineation process locates a characteristic point which was not annotated. A false negative is considered when the delineation process fails to locate the annotated fiducial point within the above mentioned tolerance of 150 ms. Positive predictive value could not be calculated, as noted in [13]: when there is no annotation it is not possible to determine whether the cardiologist considered that there was no waveform to annotate or was not confident in annotating it (perhaps because of the noise level). Nevertheless, for points other than the QRS delineation, *P*^+ ^was calculated under the assumption that an absent mark in the annotated beat means that there is no waveform. As a result, the calculated *P*^+ ^can be interpreted as a lower limit (*P*^+^*min*) of the actual one.

## Results

### QRS detection

Table 4 and Table 5 show the QRS detector performance on MITDB and EDB databases, respectively. Results are compared to previous studies. As in [13] segments with ventricular flutter in record 207 of MITDB (for an overall length of approximately 2 min 20 s) and those marked as unreadable (in the pertaining annotation file) in EDB, were excluded.

**Table 4:** Comparison of QRS Detection Performance with Published Methods (First ECG Channel of MITDB)

QRS Detector	# annotations	FP	FN	*Se *[%]	*P*^+ ^[%]
This work	109010	148	252	99.77	99.86
Martinez *et al. *[13]	109428	153	220	99.80	99.86
Ghaffari *et al. *[18]	109428	129	101	99.91	99.88
Aristotle [25]	109428	94	1861	98.30	99.91
Li *et al. *[19]	104182 (*)	65	112	99.89	99.94
Afonso *et al. *[26]	90909	406	374	99.59	99.56
Bahoura *et al. *[20]	109809 (*)	135	184	99.83	99.88
Lee *et al. *[27]	109481	137	335	99.69	99.88
Hamilton and Tompkins [28]	109267	248	340	99.69	99.77
Pan and Tompkins [23]	109809 (*)	507	277	99.75	99.54
Poli *et al. *[29]	109963	545	441	99.60	99.50
Moraes *et al. *[30]	N/R	N/R	N/R	99.22	99.73
Hamilton [31]	N/R	N/R	N/R	99.80	99.80

Inspired by [13], Table 2

(*) a discrepancy was found in the original publication between reported total and record-by-record count

N/R: not reported

**Table 5:** Comparison of QRS Detection Performance on the European ST-T Database (EDB)

QRS Detector	# annotations	FP	FN	*Se *[%]	*P*^+ ^[%]
This work	788050	3511	1483	99.81	99.56
Martinez *et al. *[13]	787103	4077	3044	99.61	99.48
Aristotle [25]	787103	10405	38635	95.09	98.63

Inspired by [13], Table 2

### ECG delineation

ECG delineation results are shown in Table 6, where they are also compared to the ones obtained in previous studies. The results reported by Ghaffari *et al. *in [18] are not included in the table because the number of leads used for detection was not stated, nor was the number of annotated beats; it is also unclear the extent to which the authors used third party annotations for validation of their algorithm on the QT Database. The accepted *two-standard-deviations *2*σCSE *tolerance, defined by the Common Standards for Electrocardiography (CSE) working party in [24] based on measurements made on different experts annotations, is also reported in the bottom row of the table. Table 7 shows inter-cardiologist annotations variability calculated on the QTDB records that were annotated by two different cardiologists. Unfortunately, only eleven records include double annotations, and only for QRS and T wave, not for P wave.

**Table 6:** Comparison of Delineation Performance with Published Methods (QT Database)

Method	Param	P onset	P peak	P offset	QRS onset	QRS offset	T peak	T offset
	# annot	3194	3194	3194	3623	3623	3542	3542
This work	*Se *[%]	98.15	98.15	98.15	100	100	99.72	99.77
	*P*^*+*^_*min *_[%]	91.00	91.00	91.00	N/A	N/A	97.76	97.76
	*m ± s*	-4.5 ± 13.4	-4.7 ± 9.7	-2.5 ± 13.0	-5.1 ± 7.2	0.9 ± 8.7	-0.3 ± 12.8	1.3 ± 18.6

Martinez *et al. *[13]	*Se *[%]	98.87	98.87	98.75	99.97	99.97	99.77	99.77
	*P*^*+*^_*min *_[%]	91.03	91.03	91.03	N/A	N/A	97.79	97.79
	*m ± s*	2.0 ± 14.8	3.6 ± 13.2	1.9 ± 12.8	4.6 ± 7.7	0.8 ± 8.7	0.2 ± 13.9	-1.6 ± 18.1

Laguna *et al. *[32]	*Se *[%]	97.70	97.70	97.70	99.92	99.92	99.00	99.00
	*P*^*+*^_*min*_[%]	91.17	91.17	91.17	N/A	N/A	97.74	97.71
	*m ± s*	14.0 ± 13.3	4.8 ± 10.6	-0.1 ± 12.3	-3.6 ± 8.6	-1.1 ± 8.3	-7.2 ± 14.3	13.5 ± 27.0

Boichat *et al. *[16] (*)	*Se *[%]	99.87	99.87	99.91	99.97	99.97	99.97	99.97
	*P*^*+*^_*min *_[%]	91.98	92.46	91.70	98.61	98.72	98.91	98.50
	*m ± s*	8.6 ± 11.2	10.1 ± 8.9	0.9 ± 10.1	3.4 ± 7.0	3.5 ± 8.3	3.7 ± 13.0	-2.4 ± 16.9

2σ_CSE _Tolerance [24]		10.2	-	12.7	6.5	11.6	-	30.6

Partially inspired by [13], Table 3

(*) 16-bit integer implementation. No. annotations not reported. *Se *and *P*^*+*^_*min *_use 320 ms window

N/A: not applicable, N/R: not reported

**Table 7:** Inter-Cardiologist Annotation Variability on QTDB (Annotation Files: q1c vs. q2c)

	# matched annotations	Mean Error ± SD [ms]
Q onset	360	-3.12 ± 14.06
T peak	359	-0.28 ± 26.24
T offset	359	-2.99 ± 39.60

## Discussion

The proposed algorithm performs online QRS detection as well as P, QRS, T waves delineation. Unlike previous DWT based methods [13], [16], [19], the present only uses two scales (2^2^, 2^3^), for both QRS detection and ECG delineation. The QRS detection showed an excellent performance on the MIT-BIH Arrhythmia Database, achieving a sensitivity of 99.77% and a positive predictive value of 99.86% on 109010 annotated beats, and on the European ST-T Database, achieving a sensitivity of 99.81% and a positive predictive value of 99.56% on 788050 annotated beats. Sensitivity and positive predictive value reported for the ST-T database are the highest among previous works, as shown in Table 5.

The validation on the QT Database showed very good performance in P, QRS, T waves delineation. The mean error (*m*) and the average standard deviation (*s*) were comparable to the ones obtained by other WT-based delineators, as shown in Table 6. Mean error (*m*) was lower than 6 ms (1.5 samples, at F_s _= 250 samples/s) for all characteristic points, whereas the average standard deviation (*s*) was around 8 ms (2 samples) for QRS delineation, and 12 ms (3 samples) for P wave and T peak delineation. Relatively high values of *s *in T wave delineation are present in all algorithms, and may be caused by the difficulty in determining the exact fiducial points as confirmed by the large inter-cardiologist annotation variability, especially for T offset as shown in Table 7.

Comparing the average standard deviation (*s*) with the *2σ*_*CSE *_tolerances, the condition *s < σ*_*CSE *_(referred to in [13] as "strict criterion") is met for P peak, QRS offset, T offset, whereas the condition *s < 2σ*_*CSE *_(referred to in [13] as "loose criterion") is not met for any of the characteristic points. However, the "strict criterion" is not met by any methods, as shown in Table 6.

Sensitivity and positive predictive value of the ECG delineator for P, QRS, T waves were comparable to the values reported by others, as shown in Table 6. However, it shall be noted that the width of the search window adopted in the computation of true positives (TP) is not the same for all methods. In [13] the window width was not reported, in [16] it was set to 320 ms. In the present work, the window width was set to 150 ms. As a result, *Se *sand *P*^*+*^_*min *_may not be comparable across different methods.

Previous DWT-based methods [13], [16], compute the adaptive thresholds in QRS detection *ε*^*k*^_*QRS *_based on the *root mean square *(RMS) of *d*^*k*^_*n *_coefficients at the scales of interest. In [13] RMS is computed over N = 2^16 ^samples excerpts, for the first three scales (2^1^, 2^2^, 2^3^). In [16] RMS is emulated over N = 2^9 ^samples excerpts for the first four scales. RMS is computationally demanding, as it requires squaring and summing N coefficients and calculating a square root. Although the square root was emulated in [16], a considerable amount of computations is required for squaring large data excerpts. In the present method, which uses only two scales, all thresholds are calculated from few (local) coefficients, which dramatically reduces the computational effort. In particular, the computation of *ε*^*2*^_*QRS *_by (5.3) only requires N = 4 data-points, compared to N = 2^9 ^in [16] and N = 2^16 ^in [13], and this computation does not require squaring as in RMS. This observation also applies to *ε*^*3*^_*QRS*_. Moreover, all thresholds are expressed in the linear form of *(A·v)/2*^*B*^, where *v *is an integer variable (or the sum of integer variables), *A *and *B *are positive constant integer values. Thus all thresholds can be computed by elementary shift and add operations.

The ECG data used in this work were either originally sampled at 250 samples/s or resampled accordingly. Although many ECG front-end devices currently on the market offer data streams at 250 samples/s or 256 samples/s, there may be devices that provide a fixed sample rate which is significantly different from 250 samples/s. In order to preserve an integer linear algebra implementation in these cases, depending on the sample rate different scales of the DWT filter bank (2) may be used, or the filter bank itself may need to be redesigned, either by using a different degree of the spline smoothing function *θ(t)*, or different scaling and wavelet functions.

## Conclusions

In this paper, a WT-based single-lead ECG delineation algorithm, designed for online 32-bit integer linear algebra processing, with shift/add operations replacing multiplications and divisions, was presented. The algorithm complies with a sample resolution up to 24-bit/sample without any assumptions on the amplitude resolution of the ECG signal.

The algorithm detects the QRS-complex, delineates the onset, dominant peak, and offset of the mono- or bi-phasic P wave, the onset and offset of the QRS-complex, the dominant peak and offset of the mono- or bi-phasic T wave.

The QRS detector achieved excellent performance on the MIT-BIH Arrhythmia database (*Se *= 99.77%, *P*^+ ^= 99.86%, 109010 annotated beats) and on the European ST-T Database, (*Se *= 99.81%, *P*^+ ^= 99.56%, 788050 annotated beats).

The proposed algorithm also exhibited very good accuracy in P, QRS, T delineator on QT Database, where the mean error between automatic and manual annotations was lower than 1.5 samples for all the characteristic points, and the associated average standard deviations were comparable to the ones reported from previous methods. However, the QTDB database contains a limited number of annotations, which makes the validation of an automatic ECG delineator not comprehensive.

Based on the results achieved on standard databases, the proposed algorithm exhibits reliable QRS detection as well as accurate ECG delineation. Reliability and accuracy are close to the highest among the ones obtained in other studies, in spite of a simplified structure built on integer linear algebra which makes the proposed algorithm a suitable candidate for online QRS detection and ECG delineation under strict power constraints and limited computational resources, such as in wearable devices for long-term non-diagnostic ambulatory monitoring.

## Competing interests

The authors declare that they have no competing interests.

## Authors' contributions

LYD designed and implemented the algorithm, carried out validation on the cited databases and participated in manuscript preparation and revisions. LC supervised manuscript preparation and revisions. All authors read and approved the final manuscript.
